# Treatment of infections caused by carbapenem-resistant *Acinetobacter baumannii*

**DOI:** 10.3389/fcimb.2024.1395260

**Published:** 2024-07-18

**Authors:** Siqin Zhang, Lingfang Di, Yan Qi, Xiang Qian, Siwei Wang

**Affiliations:** ^1^ Department of Clinical Laboratory, Hangzhou Traditional Chinese Medicine Hospital Affiliated to Zhejiang Chinese Medical University, Hangzhou, China; ^2^ Department of Clinical Laboratory, Tongxiang First People’s Hospital, Tongxiang, Zhejiang, China; ^3^ Panvascular Diseases Research Center, The Quzhou Affiliated Hospital of Wenzhou Medical University, Quzhou People’s Hospital, Quzhou, China

**Keywords:** CRAB infection, durlobactam, cefiderocol, bacteriophages, combination treatment

## Abstract

Patients with severe carbapenem-resistant *Acinetobacter baumannii* (CRAB) infections currently face significant treatment challenges. When patients display signs of infection and the clinical suspicion of CRAB infections is high, appropriate treatment should be immediately provided. However, current treatment plans and clinical data for CRAB are limited. Inherent and acquired resistance mechanisms, as well as host factors, significantly restrict options for empirical medication. Moreover, inappropriate drug coverage can have detrimental effects on patients. Most existing studies have limitations, such as a restricted sample size, and are predominantly observational or non-randomized, which report significant variability in patient infection severity and comorbidities. Therefore, a gold-standard therapy remains lacking. Current and future treatment options of infections due to CRAB were described in this review. The dose and considerable side effects restrict treatment options for polymyxins, and high doses of ampicillin-sulbactam or tigecycline appear to be the best option at the time of initial treatment. Moreover, new drugs such as durlobactam and cefiderocol have substantial therapeutic capabilities and may be effective salvage treatments. Bacteriophages and antimicrobial peptides may serve as alternative treatment options in the near future. The advantages of a combination antimicrobial regimen appear to predominate those of a single regimen. Despite its significant nephrotoxicity, colistin is considered a primary treatment and is often used in combination with antimicrobials, such as tigecycline, ampicillin-sulbactam, meropenem, or fosfomycin. The Infectious Diseases Society of America (IDSA) has deemed high-dose ampicillin-sulbactam, which is typically combined with high-dose tigecycline, polymyxin, and other antibacterial agents, the best option for treating serious CRAB infections. A rational combination of drug use and the exploration of new therapeutic drugs can alleviate or prevent the effects of CRAB infections, shorten hospital stays, and reduce patient mortality.

## 1 Introduction

*Acinetobacter baumannii* (*A. baumannii*) is a critical nosocomial pathogen that can acquire new antibiotic resistance genes, resist therapeutic medications, and avoid host immunity. Recently, the resistance rate of *A. baumannii* to all customarily used antibacterial agents has rapidly increased, a substantial proportion of which are carbapenem-resistant *A. baumannii* (CRAB). Carbapenem resistance rates exceed 30%–90% in Asia, Eastern Europe, and Latin America (Wong et al., 2017; O'Donnell et al., 2021; Seifert et al., 2022; Shields et al., 2023). Natural and acquired resistance to *A. baumannii* greatly limits the medication regimen for CRAB infections. Numerous drug resistance mechanisms of this pathogen, including biofilm formation, efflux pumps, acquisition of drug resistance, and modification of the outer membrane, are the major reasons for treatment difficulty. Worldwide, CRAB infections rank fourth in terms of antibiotic resistance (Murray et al., 2022). Hospital-acquired pneumonia (HAP) and bloodstream infections (BSI) are the most frequent CRAB infections, with mortality rates as high as 45%–70% (Bassetti et al., 2021; Pascale et al., 2021; Falcone et al., 2022). In addition to its association with concomitant diseases in patients, the treatment options available for CRAB infections are limited and preferred benchmark therapeutics are lacking (Mazzitelli et al., 2023). CRAB is listed as a critical priority on the World Health Organization list of bacteria that require new antibiotic treatments (Tacconelli et al., 2018).

Experts have still not reached a consensus on the optimal treatment of CRAB infections owing to the limited number of antibiotics and multiplicity of host factors. These conditions have forced clinicians to consider combination drug regimens and reactivate older drugs with suboptimal pharmacokinetics. Newly approved drugs and alternatives with therapeutic potential are under consideration, and various guidelines have provided different recommendations (Mazzitelli et al., 2023). Moreover, high doses of ampicillin-sulbactam or tigecycline appear to be the best option at the time of initial treatment, and polymyxins have limited treatment options because of their required high dose and significant side effects. The advantages of a combined antimicrobial regimen appear to predominate those of a single regimen. Multiple authoritative guidelines, including the European Society of Clinical Microbiology and Infectious Diseases (ESCMID) and the Infectious Diseases Society of America (IDSA) antimicrobial resistance (AMR) treatment guidelines, recommend selecting at least two antibiotics for anti-infective therapy when treating patients with severe CRAB infections (Paul et al., 2022; Tamma et al., 2022a; Zeng et al., 2023). Colistin is considered the primary treatment and is often used in combination with antimicrobials, such as tigecycline, sulbactam, meropenem, or other antibiotics. New treatment options, such as durlobactam and cefiderocol, have recently emerged and have shown considerable therapeutic potential. In addition, alternative treatment options such as bacteriophages have made significant progress.

**Figure 1:**
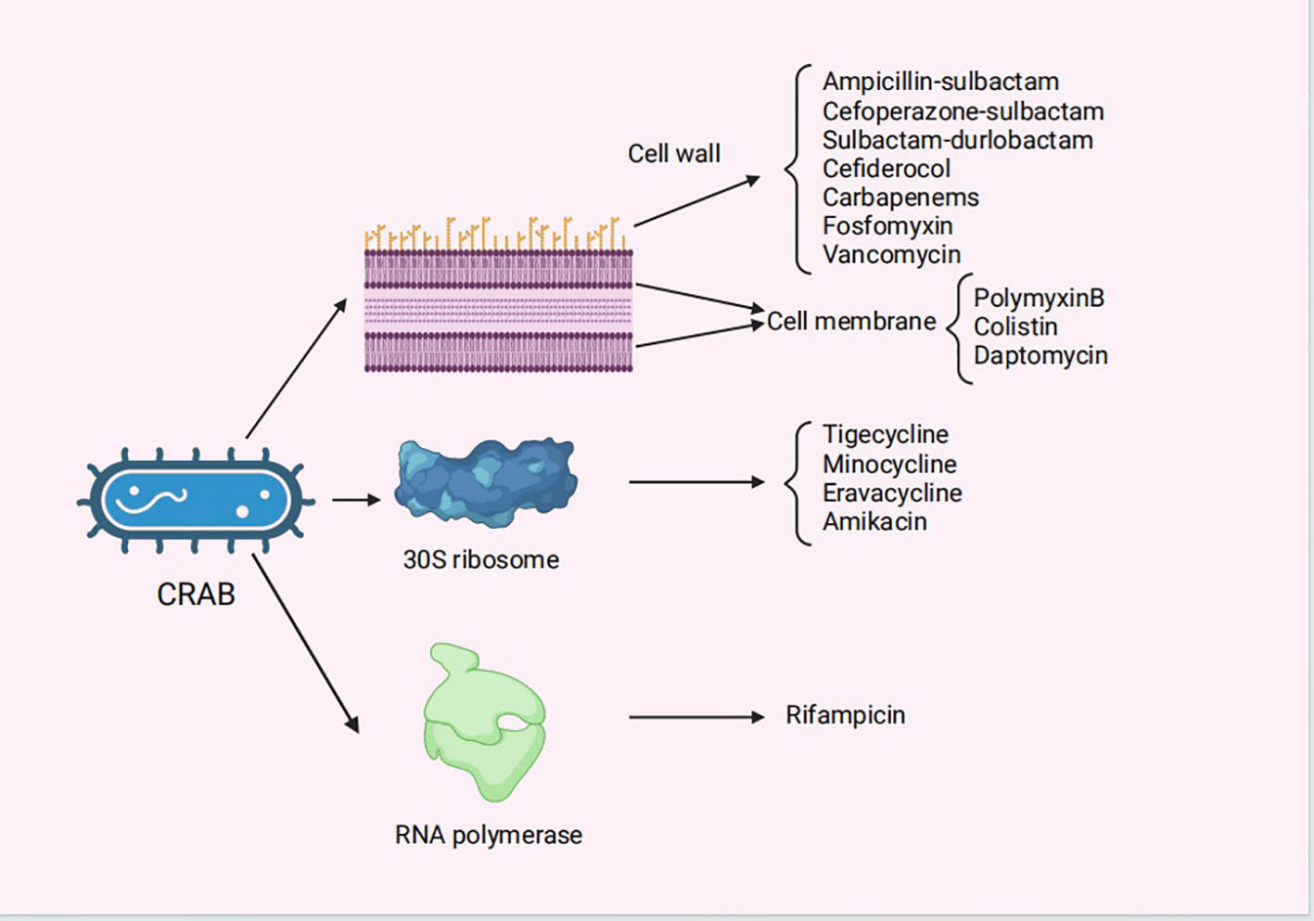
The mechanisms of action for the antibiotics for the treatment of CRAB infections. CRAB, carbapenem-resistant *Acinetobacter baumannii.*.

In this review, current and future therapeutic approaches of infections caused by CRAB are described, with the relevant information summarized in **Table 1**, **Figure 1**.

**Table 1:** The action mechanisms, promise, perils, attentions and common combinations of antibacterial agents for the treatment of CRAB infections.

Agent	Action Mechanism	Promise	Perils	Attentions	Common Antibacterial Combinations
β-lactams–β-lactamase inhibitors					
Ampicillin-sulbactam, cefoperazone-sulbactam	• Protect the beta-lactam ring from hydrolysis by enzymes • Bind to penicillin binding proteins2a	• Have good antibacterial activity against acinetobacter • Expands the antibacterial spectrum	• High drug resistance rate of sulbactam alone • Hepatotoxicity	• High-dose (9g q8h) are required	• High-dose ampicillin-sulbactam combined with another active agent such as high-dose tigecycline, polymyxins, minocycline, etc (Bartal et al., 2022; Zeng et al., 2023)
Sulbactam/durlobactam	• Can restore the susceptibility to sulbactam in *A. baumannii* strains producing class A, C, and D β-lactamases	• Approved in May 2023 by the USA FDA to treat hospital-acquired and ventilator-associated bacterial pneumonia • A valuable option in critically ill patients	• Very little evidence in bloodstream infections • Use as a monotherapy or in combination with other agents is inconclusive	• Not to use this antibiotic for non-severe infections or when less potent options are appropriate	• Combined with imipenem-cilastatin combined with cefiderocol or meropenem (Zaidan et al., 2021; Holger et al., 2022; Kaye et al., 2023; Tiseo et al., 2023)
β-lactams					
Cefiderocol	• Can quickly penetrate the outer cell membrane of gram-negative bacteria • Can resist the hydrolysis of most extended-spectrum beta-lactamases, class A, B, and D carbapenases, and class C cephalosporinases • Stable against hydrolysis by serine β-lactamases and metallo-β-lactamases	• The first siderophore cephalosporin approved by the USA FDA to treat carbapenem-resistant gram-negative pathogens • Potent *in vitro* activity against CRAB isolates from • A variety of infection sources(susceptibility rates of >90%) • Adverse events are very infrequent	• The effectiveness data from current clinical trials are contradictory • The difference of 30-day mortality is lower was confirmed in bloodstream infections but not in ventilator-associated pneumonia	• Used for infections in which other antibiotics resulted in treatment failure and only in combination with other antibiotics that are active *in vitro*(the IDSA and ESCMID guidelines)	• Combined with fosfomycin, tigacycline and colistin as rescue treatment for severe CRAB infections (Bavaro et al., 2021; Mabayoje et al., 2021; Tan et al., 2022; Karruli et al., 2023)
Carbapenems					
Imipenem, Meropenem, Panipenem, Biapenem, etc	• Inhibit penicillin-binding proteins, thus hindering the synthesis of cell wall mucopepidin	• Widely used to treat serious infections	• Drug-resistant strains have erupted in the world • None of the new β-lactam–β-lactamase inhibitor combinations are active against CRAB	• Continuous infusion of high-dose meropenem and therapeutic drug monitoring • Do not advocate for the treatment of CRAB infections(IDSA)	• Combined with meropenem, polymyxin B and ampicillin/sulbactam for the treatment of CRAB pneumonia, with either meropenem plus minocycline or combination treatment with cefiderocol as alternative options (SIDP) (Abdul-Mutakabbir et al., 2021)
Polymyxins					
Polymyxin B, Colistin	• The positively charged free amino group can bind to the negatively charged phosphate group in the phospholipid of the cell membrane	• Less deaths • Less clinical treatment failure • The synergistic effect with linezolid and vancomycin	• Considerable nephrotoxic effect • Hyponatremia, • Hypomagnesemia • Hypokalemia • Hypophosphatemia	• Most of the published literature focusing on colistin • Limited penetration into lung or urine	• Combined with tigecycline, ampicillin/sulbactam, meropenem or fosfomycin (Kengkla et al., 2018)
Tetracyclines					
Tigecycline	• Bind to the a site of 30s subunit of bacterial ribosomes • Prevent bacterial transcription and inhibits protein synthesis	• Wide antibacterial spectrum • Wide distribution • Good stability • Low toxicity of target organs • Long half-life • Excellent penetration for skin, soft tissue and osteoarticular infections	• The low plasma concentrations • Lack of established susceptibility breakpoints	• Higher tigecycline doses (200 mg loading dose followed by 100 mg every 12 hours) have been associated with improved outcomes • The clinical efficacy was associated with MIC value	• Combined with high-dose ampicillin-sulbactam, polymyxin, cefiderocol (Ni et al., 2016; Bartal et al., 2022; Zeng et al., 2023)
Minocycline	• Bind to tRNA	• With FDA approval to treat CRAB infections • Available as an oral formulation • Intravenous minocycline provided high rates of clinical success or improvement	• Susceptibility breakpoints are unclear • The lack of modern PK/PD studies and randomized controlled trials	• Combination drugs are recommended to avoid the development of resistance	• Combined with other agents such as colistin, rifampin and carbapenems (Ritchie and Garavaglia-Wilson, 2014; Greig and Scott, 2016)
Eravacycline	• Bind to the 30S ribosome subunit to inhibit bacterial protein synthesis	• Lower MICs than minocycline or tigecycline • Approved for treatment of complicated intra-abdominal infections	• No clinical breakpoints	• Limited data to treat CRAB • Use with caution in bacteremia	/
Aminoglycosides					
Amikacin	• Act on ribosomes in bacteria, inhibits bacterial protein synthesis • Destroy the integrity of bacterial cell walls	• Inhaled aminoglycosides and colistin decrease bacterial burdens	• High resistance rates (68% to 100%) and nephrotoxicity	• Limited data to treat CRAB	• Combined with colistin (Vardakas et al., 2018; Niederman et al., 2020)
Rifamycin					
Rifampicin	• Bind firmly to the beta subunit of DNA-dependent RNA polymerase and block the RNA transcription process	• A wide antibacterial spectrum	• Known toxicities • Drug-drug interactions	• Limited clinical data	• Combined with colistin (Aydemir et al., 2013; Durante-Mangoni et al., 2013)
Polyphosphates					
Fosfomycin	• Combine with bacterial cell wall synthetase	• Broad spectrum • Enzyme resistance • High efficiency • Low toxicity	• Limited supply of intravenous fosfomycin	• Limited clinical data	• Combined with colistin (Sirijatuphat and Thamlikitkul, 2014)
Glycopeptide or lipopeptide antibiotics					
Vancomycin	• Inhibit the synthesis of peptidoglycan in the bacterial cell wall	• The combination with colistin has unique potential effects	• Acute kidney injury	• Clinical applications are contradictory	• Combined with colistin (Garnacho-Montero et al., 2014; Katip et al., 2021)
Daptomycin	• Destroy bacterial cell membranes	• An option after second-line treatment has failed	• The efficacy and safety remains to be confirmed	• Limited clinical data	• Combined with colistin (Cirioni et al., 2016; Poulakou et al., 2019)

CRAB, carbapenem-resistant Acinetobacter baumannii; FDA, Food and Drug Administration; IDSA, Infectious Diseases Society of Americ; ESCMID, European Society of Clinical Microbiology and Infectious Disease; SIDP, Society of Infectious Diseases Pharmacists; MIC, minimum inhibitory concentration.

## 2 Current treatment options for CRAB infections

### 2.1 β-lactams–β-lactamase inhibitors

#### 2.1.1 Ampicillin-sulbactam and cefoperazone-sulbactam

β-lactamase inhibitors, including clavulanic acid, sulbactam, and tazobactam, protect the β-lactam ring. Non-β-lactam β-lactamase inhibitors have a wide antibacterial spectrum; however, their effect on class B carbapenemases is not ideal, and there are no curative effects on CRAB infections by new drugs such as avibactam, relebactam, and vaborbactam (Bartal et al., 2022).

Sulbactam was approved in the 1980s and has since been used as a clinical anti-infective agent. It is a competitive β-lactamase inhibitor, whose action is irreversible, that can bind to PBPs2a when administered in large doses (Penwell et al., 2015). When combined with β-lactam antibiotics, sulbactam not only has good antibacterial activity against *Acinetobacter* but also expands the antibacterial spectrum. Because other inhibitors in the same class do not exhibit this activity, the additional role of sulbactam is particularly prominent in the treatment of severe CRAB infections (Bartal et al., 2022). The resistance mechanism of *A. baumannii* to sulbactam is primarily associated with the downregulation of penicillin-binding protein 2 expression and the production of β-lactamase containing the TEM-1 resistance gene. The two most common sulbactam combinations are ampicillin-sulbactam and cefoperazone-sulbactam. Ampicillin-sulbactam has a wide range of applications and is used worldwide, whereas cefoperazone-sulbactam is commonly used in several Asian nations. The overall response rate of high-dose ampicillin-sulbactam (9 g/8 h) for treating ventilator-associated pneumonia caused by CRAB was 76.9%, thereby demonstrating an efficacy and safety similar to those of polymyxin (Betrosian et al., 2008).

However, due to the recently determined high antibiotic resistance rate of using sulbactam alone, the combination of sulbactam and β-lactam antibiotics with other antibiotics has gradually become an increasingly popular choice for treating patients with CRAB infections. In clinical practice, antibiotics such as polymyxin, tigecycline, minocycline, and doxycycline are typically selected based on antibiotic susceptibility testing (Zeng et al., 2023). In South Korea, a multicenter study on CRAB treatment strategies revealed that ampicillin-sulbactam treatment exhibited the lowest 7-day mortality rate (13.0%) and reduced the 28- and 7-day mortality rates in patients (Seok et al., 2021). By comparing the curative effects of meropenem/ampicillin-sulbactam with those of meropenem/colistin in patients with severe pneumonia caused by CRAB, Khalili et al. suggested that ampicillin-sulbactam was an efficient treatment option for CRAB infections (Khalili et al., 2018). A systematic review reported that high-dose sulbactam (more than 6g per day) combined with levofloxacin or tigecycline had an increased curative effect and that high-dose ampicillin-sulbactam (more than 18g per day) combined with other drugs effectively reduced the mortality of patients with severe CRAB infections (Liu et al., 2021). Furthermore, in a retrospective study of bloodstream infections caused by CRAB, the 28-day mortality rate of patients treated with cefoperazone-sulbactam was lower than that of patients treated with tigecycline (29.3% vs. 51.9%, respectively). The mortality rate of patients treated with cefoperazone-sulbactam combined with imipenem-cilastatin was considerably lower than that of patients treated with cefoperazone-sulbactam alone (Niu et al., 2019). Thus, these data show that sulbactam-containing combination therapies have clinical benefits. The currently accepted treatment modality is ampicillin-sulbactam as a monotherapy. Except for patients with a penicillin allergy, combining other antibiotics with ampicillin-sulbactam should be considered based on susceptibility results. For intolerance or toxicities that prevent using higher dosages or for mild infections, the typical dose of sulbactam is 3 g every 4 h, and for ampicillin-sulbactam-resistant CRAB, the dose is 9 g every 8 h. Each dose is administered over 4 h or with a 27 g continuous infusion over 24 h (Bartal et al., 2022).

#### 2.1.2 Sulbactam/durlobactam

Sulbactam/durlobactam (SUL/DUR) is a composite antibiotic of both a β-lactamase and non-β-lactam β-lactamase inhibitor. Sulbactam (SUL) is a semisynthetic penicillanic acid with weak intrinsic inhibitory activity against class A serine β-lactamases (including KPC-2,CTX-M-15, TEM-1, and SHV-5), but no inhibitory activity against class C and D β-lactamases (Shapiro, 2017). Durlobactam (DUR) is a non-β lactam diazabicyclooctanone inhibitor, and although it has no inhibitory activity against class B metallo-β-lactamases, it has inhibitory activity against class A, C, and D serine β-lactamase (Shapiro, 2017). Therefore, SUL/DUR may be used against *A. baumannii*, which produces class A, C, and D β-lactamases. The frequency of spontaneous *in vitro* resistance to SUL/DUR is low, and the most common mutation found in stable mutants is in the *ftsI* gene (the target gene encoding SUL PBP3) (Karruli et al., 2023). Current data show that mice treated with SUL/DUR can resist CRAB both *in vitro* and *in vivo* (Findlay et al., 2022). The FDA approved SUL/DUR in May 2023 for treating ventilator-associated and hospital-acquired bacterial pneumonia in patients over 18 years of age caused by sensitive *A. baumannii* isolates (https://www.accessdata.fda.gov/drugsatfda_docs/label/2023/216974Orig1s000Correctedlbl.pdf) (US Food and Drug Administration, 2023).

Kaye et al. compared the safety and efficacy of colistin versus SUL/DUR in the treatment of severe bloodstream infection, ventilator-associated bacterial pneumonia, ventilated pneumonia, and HAP caused by CRAB in a large multinational phase 3 study, and both regimens were combined with imipenem-cilastatin. Compared to the colistin regimen, the SUL/DUR regimen had lower rates of critical adverse events, nephrotoxicity, and 28-day mortality (40% vs 49%, 13% vs 38%, 19% vs 32%, respectively), indicating that SUL/DUR was effective in reducing the mortality rate of severe CRAB infections (Kaye et al., 2023). Certain case reports have shown that SUL/DUR combined with cefiderocol or meropenem can improve the prognosis of patients with CRAB infections (Zaidan et al., 2021; Holger et al., 2022; Tiseo et al., 2023). Although current clinical case data suggest that SUL/DUR may be an excellent treatment for severe CRAB infections, the clinical evidence for this new drug is still extremely limited, particularly for bloodstream infections. Therefore, it is not conclusive whether this new antibiotic will fight CRAB as a single antibiotic or in combination with other antibiotics.

### 2.2 β-lactams

#### 2.2.1 Cefiderocol

As a drug that belongs to a new class of catechol-substituted siderophore cephalosporins, cefiderocol has zwitterionic properties and can rapidly penetrate the outer cell membrane of Gram-negative bacteria (Karruli et al., 2023). Cefiderocol is FDA approved for the treatment of CRAB, carbapenem-resistant *Pseudomonas aeruginosa*, and carbapenem-resistant *Enterobacteriales* (CRE), and it was the first siderophore cephalosporin to be approved (https://www.accessdata.fda.gov/drugsatfda_docs/label/2019/209445s000lbl.pdf) (US Food and Drug Administration, 2019). Cefiderocol is resistant to extended-spectrum β-lactamases (ESBLs), class C cephalosporinases, and class A, B, and D carbapenamases (Kollef et al., 2023). Additionally, the primary mechanism underlying drug resistance in carbapenem pathogens is the production of serine β-lactamases, and cefiderocol exhibits resistance against both serine β-lactamase and metallo-β-lactamase hydrolysis. Cefiderocol can sustain resistance to PER-1-producing *A. baumannii*, as demonstrated using *in vitro* models (Yamano et al., 2020), and the resistance to cefiderocol is correlated with reduced expressions of *pirA* and *piuA* (Karruli et al., 2023).

*In vivo* tests have confirmed that cefiderocol exhibits robust tissue permeability and favorable pharmacokinetic/pharmacodynamic characteristics in both infected patients and healthy controls. In addition, *in vitro* tests have confirmed that cefiderocol displays bactericidal activity against CRAB strains isolated from diverse clinical specimens, with an overall susceptibility rate exceeding 90% (Kollef et al., 2023). *In vivo*, resistance that emerges during treatment is present in a low proportion/sporadic cases but is less common. A multicenter cohort study, which included 107 ICU patients with CRAB infections, compared the potencies of colistin and cefiderocol and confirmed that cefiderocol had significant efficacy in patients with CRAB infections; however, this conclusion needs to be supported by large-scale clinical data (Pascale et al., 2021). A propensity score-adjusted retrospective monocentric cohort study evaluated the anti-infective efficacy of cefiderocol and colistin against blood-derived CRAB isolates and demonstrated that cefiderocol could be an effective choice for treating bloodstream infections in CRAB with restricted management alternatives (Bavaro et al., 2023). Moreover, the incidence of adverse events was lower in patients treated with cefiderocol than in those treated with colistin (Bavaro et al., 2023). An observational retrospective study to compare the efficacy of cefiderocol and colistin conducted by Falcone et al. at the University Hospital of Pisa revealed that the 30-day mortality in patients with bloodstream infections treated with cefiderocol was lower than that in those treated with colistin, and the risk of death was reduced by 60% (34% versus 55.8%). However, no such difference was observed in patients with ventilator-associated pneumonia (Falcone et al., 2022). Therefore, cefiderocol may be a valuable alternative for treating serious CRAB infections (Falcone et al., 2022). Several reports have confirmed the effectiveness of cefiderocol combined with fosfomycin, tigecycline, and colistin as rescue treatments for serious CRAB infections, and the side effects of cefiderocol are rare (Bavaro et al., 2021; Mabayoje et al., 2021; Tan et al., 2022; Karruli et al., 2023).

Certain treatment studies have reported conflicting results. For example, a study conducted at the University Hospital of Padua assessed the effectiveness, microbiological variances, and predictors of the 30-day mortality in patients with CRAB infections who were treated with cefiderocol and colistin. These findings confirmed no disparities in patient outcomes or safety between cefiderocol and colistin (Mazzitelli et al., 2023). The data also revealed that cefiderocol, when combined with other drugs, did not ameliorate the symptoms in CRAB-infected patients compared to that of colistin combined with other drugs. However, when cefiderocol is used, the role of monotherapy versus combination therapy and which drug is better when combined, remain to be clarified (Mazzitelli et al., 2023). Certain studies have shown that the risk of anti-infective treatment failure with cefiderocol monotherapy is higher than that with cefiderocol combined with other antibiotics. Thus, a combination regimen can mitigate selective antibiotic resistance in most cases (Band et al., 2019; Falcone et al., 2022). Mazzitelli et al. believed that cefiderocol should only be used in certain cases, such as when other options have proven ineffective or in clinical situations where the risk of toxicity caused by colistin is unacceptable (Mazzitelli et al., 2023).

Because the effectiveness data from current clinical trials are contradictory, the IDSA and ESCMID guidelines propose that the cefiderocol combination regimen must be combined with an *in vitro-*effective antibiotic, and this drug should be selected when other antibiotics have failed (Paul et al., 2022; Tamma et al., 2023). International consensus guidelines recommend a regimen of 2 g every 8 h infused over 3 h. Although cefiderocol exhibits superior *in vitro* activity and tolerability in most studies, prospective clinical data in patients with CRAB infections are insufficient to support its widespread application, and the clinical benefits of combination therapy have not been established in prospective studies.

### 2.3 Carbapenems

Carbapenem antibiotics have the widest antibacterial range and greatest antibacterial action in atypical β-lactam antibiotics. The mechanism of action involves the inhibition of penicillin-binding proteins, thus hindering the synthesis of cell wall mucopepidin and causing bacterial cell wall defects and body expansion. This results in bacterial plasma osmotic pressure changes, dissolution, and killing. Currently, carbapenem antibiotics used in clinical practice include imipenem, meropenem, biapenem, and panipenem. This class of antibiotics is widely prescribed for serious infections such as HAP, complex abdominal infections, and bloodstream infections, and carbapenems were once considered effective drugs for treating *A. baumannii* infections. Recently, carbapenems have been prescribed for diseases caused by multidrug-resistant *A. baumannii*; however, drug-resistant bacterial strains have emerged worldwide, causing difficulties in the treatment and control of infections. For instance, isolates of hospital-acquired *A. baumannii* infections in Europe have a carbapenem resistance rate of up to 80%, and the primary mechanism of carbapenem resistance in *A. baumannii* is the production of carbapenemases (classes D, A, and B). Additionally, a decrease in membrane permeability and the upregulation of efflux pumps are important mechanisms (Falcone et al., 2022). New β-lactam–β-lactamase inhibitor combinations, such as imipenem/relebactam, meropenem/vaborbactam, aztreonam/avibactam, ceftazidime/avibactam, and ceftolozane/tazobactam, have no anti-infection effects on CRAB isolates (Hsueh et al., 2019; Mueller et al., 2019; Karakonstantis et al., 2020).

Studies have proposed that if meropenem is selected for managing CRAB infections, its high-dose continuous infusion and therapeutic drug monitoring are required to obtain more advantageous medical results and an antibiotic exposure higher than the target MIC value of these antibiotics (Liebchen et al., 2020). In a multicenter study performed in a highly endemic area of South Korea, carbapenem and colistin combination therapy reduced the 7-day mortality rate in CRAB infections (Seok et al., 2021). In the guidance document from the IDSA, the combination regimen for treating CRAB infections does not recommend the inclusion of carbapenems (Tamma et al., 2022a). A combination of meropenem, ampicillin/sulbactam, and polymyxin B for treating pneumonia caused by CRAB isolates and meropenem plus minocycline or a combination of cefiderocol as alternative selections were recommended by the Society of Infectious Diseases Pharmacists (SIDP) (Abdul-Mutakabbir et al., 2021). This suggests that the treatment modality should not include carbapenems; if chosen, there is a clinical preference for carbapenems as a third drug in the combination regimen. The dosage varies depending on the drug, with a recommended dosage of 500 mg every 6 h infused over 3 h for imipenem, and 2 g every 8 h infused over 3 h for meropenem.

### 2.4 Polymyxins

Polymyxins are polypeptide antibiotics extracted from the culture medium of *Bacillus polymyxis* that are classified into five types: A, B, C, D, and E. Polymyxins B and E are the most commonly used in medical practice and have anti-CRAB effects; however, most studies have focused on polymyxin E, also known as colistin. Its mechanism of action involves the positively charged free amino group in the drug molecule binding to the negatively charged phosphate group in the phospholipids of the cell membrane of Gram-negative pathogens. It can competitively replace calcium and magnesium ion channels, destroy the permeability of the outer membrane, and cause important substances, such as amino and nucleic acids, to leak out in the bacteria, thus resulting in death. The development of drug resistance is associated with the upregulation of *pmrAB* in the two-component regulatory system and the mutation of *lpxC*, the lipopolysaccharide-encoding gene.

Currently, the best treatment option for infections caused by CRAB isolates has not been identified. Despite its nephrotoxic effects, colistin, in combination with other potent agents, such as tigecycline, ampicillin/sulbactam, meropenem, and fosfomycin, is an important management option (Kengkla et al., 2018). It is widely used for treating conditions such as bloodstream, lung, and urinary tract infections. In the case of central nervous system infections, an intrathecal or intraperitoneal injection may be considered, and the dosage should be in accordance with international consensus guidelines (Bartal et al., 2022). Several retrospective studies have explored the anti-infective efficacy of a colistin-combined regimen versus a tigecycline-combined regimen for CRAB. These studies have reported that the colistin-combined regimen had lower mortality and clinical treatment failure rates than that of the tigecycline-combined regimen; however, it had a higher incidence of side effects related to nephrotoxicity (Liang et al., 2018; Park et al., 2021; Russo et al., 2021). Other studies have demonstrated that in treating ventilator-associated pneumonia caused by CRAB isolates, colistin combined with rifampin has lower clinical mortality and a stronger bactericidal effect on isolates than colistin monotherapy (Aydemir et al., 2013; Durante-Mangoni et al., 2013). Moreover, a colistin and rifampin combination regimen has been successfully used to treat pneumonia and meningitis caused by CRAB (Rodríguez et al., 2012; Park et al., 2019). Polymyxin combined with meropenem and high-dose ampicillin-sulbactam is another promising combination that has been successfully used to treat ventilator-associated pneumonia caused by CRAB (Lenhard et al., 2017; Assimakopoulos et al., 2019). Notably, colistin combined with drugs (like linezolid and vancomycin) that are inactive against Gram-negative pathogens exert a synergistic effect, indicating that colistin could exercise sub-inhibitory permeability, thereby enabling more of the other drugs to enter the pathogenic bacteria (Bae et al., 2016). Additionally, the loading dose of colistin may benefit patients. For example, the results of a retrospective study involving 383 patients indicated that on day 30 of treatment, the survival rate, clinical efficacy, and pathogen eradication rate were significantly higher in patients receiving a loading dose of colistin than in those receiving a non-loading dose of colistin; however, the close monitoring of renal function was necessary (Katip and Oberdorfer, 2021). Another prospective study confirmed that a high loading dose of colistin was safe and effective for treating multi-drug resistant (MDR) *A. baumannii* (Katip et al., 2019). Katip et al. conducted a retrospective study of cases spanning seven years and confirmed the combination of meropenem with a loading dose of colistin regimen for treating CRAB infections (Katip et al., 2022). However, this conclusion is neither absolute nor contrary. For example, the application of a loading dose of colistin in patients with cancer or ventilator-associated pneumonia did not improve efficacy but significantly increased renal toxicity (Alp et al., 2017; Katip et al., 2017). Further clinical evidence is required to confirm the benefits and risks of colistin loading.

Owing to the high number of comorbidities in patients with CRAB infections, the safety of the drugs used for anti-infective remedies is particularly important. Notably, drug side effects are primarily observed in patients receiving colistin regimens (Falcone et al., 2022; Bavaro et al., 2023). A review of 237 controlled studies revealed that patients treated with polymyxin had a higher incidence of nephrotoxicity than those who were not (Wagenlehner et al., 2021). Colistin use can also lead to other adverse effects such as hyponatremia, hypomagnesemia, hypokalemia, and hypophosphatemia (Moni et al., 2020; Kollef et al., 2023). In addition, the permeability of polymyxins to the lungs and urinary system is insufficient (Abdul-Mutakabbir et al., 2021). Notably, polymyxin B has better pharmacokinetics and lower nephrotoxicity than colistin; however, most studies on polymyxins have focused on colistin. Consequently, clinical data regarding the efficacy of polymyxin B as a treatment option are lacking (Tsuji et al., 2019; Karakonstantis et al., 2020).

### 2.5 Tetracyclines

#### 2.5.1 Tigecycline

Tigecycline is a glycyltetracycline-based antibiotic. Its mechanism of action is similar to that of tetracycline agents. By connecting to the 30s subunit of bacterial ribosomes, it prevents bacterial transcription and inhibits protein synthesis, thereby inhibiting bacterial proliferation. Tigecycline has a wide antibacterial spectrum, wide distribution, good stability, low dosage, low toxicity to target organs, easy development of drug resistance, long half-life, and is widely usable, rendering it a choice for treating serious infections, particularly against most refractory pathogens with strong antibacterial effects (Paul et al., 2022; Tamma et al., 2022a; Tamma et al., 2022b). Moreover, tigecycline can achieve high concentrations in several tissues of the body. For example, the concentration of the drug in the lungs is two times higher than that in the serum (Zeng et al., 2023). Tigecycline has been suggested as an anti-infective medication for patients with abdominal and pulmonary infections caused by CRAB and CRE; however, numerous strains remain resistant to tigecycline, and the mechanism of resistance is related to the high expression of the effector pump gene (AdeABC) in MDR *A. baumannii* (Paul et al., 2022; Tamma et al., 2022a; Tamma et al., 2022b).

Oliveira et al. compared the efficacy of colistin and tigecycline in patients with osteomyelitis caused by CRAB isolates. Their data confirmed that tigecycline was safer than colistin for the treatment of osteomyelitis; however, no such discrepancy was observed after one year of follow-up (Oliveira et al., 2020). For infected patients with poor renal function, a combination of polymyxins is considered acceptable (Abdul-Mutakabbir et al., 2021). Impact therapy with a 200 mg loading dose followed by 100 mg every 12 h may enhance the curative effect in infected patients (De Pascale et al., 2020; Zha et al., 2020). This suggests that the conventional doses for bloodstream or lung infections may be inadequate. Because high-dose tigecycline (200mg once, then 100mg every 12h) safely increases plasma and pulmonary concentrations, prioritizing high-dose over standard-dose tigecycline for treating severe CRAB infections may be practical.

In patients with pneumonia or bacteremia, all-cause mortality was higher with tigecycline monotherapy than with other regimens (Jung et al., 2017; Liang et al., 2018). As abovementioned, the tigecycline therapy regimen can reduce nephrotoxicity and other side effects, but its efficacy is worse than that of the polymyxin therapy regimen. When the tigecycline MIC ≤2 mg/L, its efficacy is comparable to polymyxin, and when the tigecycline MIC >2 mg/L, its efficacy is worse than polymyxin. Thus, the efficacy of tigecycline is closely related to the MIC values in the drug susceptibility testing (Chuang et al., 2014). A meta-analysis assessed the effect of tigecycline in the treatment of CRAB infections, revealing that patients treated with tigecycline had a higher in-hospital mortality, significantly lower CRAB clearance, and longer hospital stay than those treated with tigecycline alone. Thus, tigecycline-based treatment did not reduce patient mortality or improve clinical recovery rates. This result does not support the use of tigecycline-based treatments (Ni et al., 2016). Due to its pharmacokinetic problems, low plasma and cerebrospinal fluid concentrations, and lack of a clear susceptibility breakpoint, its use in bloodstream and central system infections remain limited (Abdul-Mutakabbir et al., 2021).

#### 2.5.2 Minocycline

Minocycline is a tetracycline derivative that was synthesized in the 1960s. It has a wide antibacterial spectrum and rapid antimicrobial activity against both Gram-negative and Gram-positive pathogenic bacteria. Minocycline is an “old drug” approved by the FDA for managing *Acinetobacter* infections; however, it lacks PK/PD research data, and its susceptibility break point is not clear (Greig and Scott, 2016; Karakonstantis et al., 2020).

In the case of noninvasive infections, minocycline has an oral preparation with the advantage of degradation. Minocycline has a good anti-infection effect on CRAB isolates *in vitro* (Noel et al., 2021). When coupled with carbapenems, colistin, or rifampin, minocycline exhibits synergistic bactericidal activity against CRAB isolates (Greig and Scott, 2016). Studies have demonstrated that intravenous minocycline injections yield a better clinical cure rate and are well tolerated in patients infected with CRAB isolates. Although one study demonstrated the possible role of intravenous minocycline in the treatment of patients infected with CRAB isolates, there are currently few relevant clinical data (Ritchie and Garavaglia-Wilson, 2014; Greig and Scott, 2016). Goff et al. treated 55 patients infected with MDR *A. baumannii* with minocycline, three with monotherapy, and 52 with minocycline in combination with other drugs, resulting in the successful treatment of 73% of patients. Although treatment with minocycline alone is effective, it is recommended that clinicians use this combination frequently to prevent the development of resistance (Goff et al., 2014). In an *in vitro* pharmacodynamic model, a regimen of polymyxin B and high-dose minocycline combined with continuous-infusion sulbactam exhibited the strongest bactericidal effect on CRAB isolates, with no regeneration or minimal resistance development (Beganovic et al., 2021). The curative effect of minocycline on CRAB is efficient; however, because of potential adverse reactions in the gastrointestinal tract and vestibular system, this regimen is not preferred. If chosen, sulbactam or a combination of sulbactam with minocycline should be considered, with a recommended dose of 200 mg administered every 12 h.

#### 2.5.3 Eravacycline

Eravacycline belongs to a new class of synthetic fluorocyclines and is not inhibited by a resistance to tetracycline efflux pumps and ribosome-protective proteins. Its MIC value is typically lower than that of tigecycline and minocycline; however, the clinical breakpoint is not clear according to the Clinical and Laboratory Standards Institute and European Committee for Antimicrobial Susceptibility Testing. This drug is approved for treating complex abdominal infections caused by sensitive pathogens, but not for the treatment of infections caused by CRAB isolates. The medication is available in both oral and intravenous forms, with an oral bioavailability of only 28%, and the recommended dose is 1 mg/kg every 12 h (Morrissey et al., 2020).

Clinical study data are currently limited regarding the use of eravacycline for the treatment of CRAB. However, compared with tigecycline, *in vitro* and *in vivo* studies have shown that eravacycline has stronger antibacterial activity against CRE and CRAB, a higher concentration in lung tissue, and fewer side effects. Whether the clinical efficacy of tigecycline is better requires further confirmation through relevant prospective studies (Corcione et al., 2022; Gavaghan et al., 2023). In a retrospective study of adults hospitalized for pneumonia with difficult-to-treat, resistant *A. baumannii*, data showed that patients treated with eravacycline had lower pathogen clearance, higher 30-day mortality, and longer mechanical ventilation than those treated with the best prior treatment. The efficacy of eravacycline was similar to that in the control group, but the efficacy in the treatment of bacteremia was not ideal; therefore, eravacycline should be used with caution (Scott et al., 2022). It is a potential candidate for treating severe CRAB infections, but further evaluation is required.

### 2.6 Aminoglycosides

#### 2.6.1 Amikacin

Amikacin exhibits *in vitro* antibacterial activity against pathogens and is another antibiotic used to treat CRAB infections. However, its high resistance rate, which can exceed 68%, and the side effects, such as renal toxicity, of aminoglycoside drugs limit its clinical application (Isler et al., 2019). Clinical data on this treatment regimen are also limited. In a multicenter study conducted in a highly endemic area of South Korea, the related clinical response was increased in patients treated with amikacin (Seok et al., 2021). In addition, patients with pneumonia caused by CRAB strains received inhaled aminoglycosides and colistin treatment, which cleared certain pathogens; however, whether this could improve clinical prognosis remains unknown (Vardakas et al., 2018; Niederman et al., 2020). The effect of this treatment modality on CRAB infections must be confirmed; thus, it is not the preferred treatment option.

### 2.7 Rifampicin

Rifampicin is a semi-synthetic antibiotic that binds to the beta subunit of DNA-dependent RNA polymerase, thereby preventing this enzyme from binding to DNA, blocking RNA transcription, and stopping DNA and protein synthesis. Rifampicin has a wide antibacterial spectrum and exhibits good antibacterial activity against *Mycobacterium tuberculosis*, nontuberculous mycobacteria, Gram-negative bacteria, and Gram-positive pathogens. Its mechanism of resistance is primarily related to the substitution of amino acids in the β-subunit of the target protein and can be caused by a single mutation in the *rpoB* gene; therefore, rifampicin alone is not recommended.

As mentioned in Section 2.4, rifampicin combined with polymyxins has a significant effect on ventilator-associated pneumonia-related mortality or microbiological responses compared to that of colistin monotherapy; however, the safety of the drug needs to be considered (Aydemir et al., 2013; Durante-Mangoni et al., 2013). A study using colistin monotherapy or colistin plus rifampicin reported that the combination treatment shortened the bactericidal time and increased bacterial clearance; however, it lacked evidence of improved efficacy and the risk of side effects, such as hepatotoxicity, caused by rifampicin (Al-Shaer et al., 2014). It is not recommended for routine clinical use in CRAB infections owing to insufficient clinical evidence, known side effects, and drug–drug interactions (Bartal et al., 2022).

### 2.8 Fosfomycin

Fosfomycin can combine with bacterial cell wall synthetase to prevent bacteria from using related substances to synthesize their cell wall, thus playing a bactericidal role, and is mostly used to treat urinary tract infections. Fosfomycin alone can easily induce drug resistance; therefore, it is often used in combination with other drugs. Recently, intravenous fosfomycin and combination treatments for CRAB infections have attracted increasing attention (Bartal et al., 2022; Marino et al., 2022).

A study including 94 patients infected with CRAB revealed that patients treated with fosfomycin plus colistin had higher pathogen clearance rates, better clinical outcomes, and lower mortality rates than those treated with colistin monotherapy (Sirijatuphat and Thamlikitkul, 2014). A prospective observational multicenter study that included 180 patients with HAP demonstrated the superiority of regimens that included fosfomycin (Russo et al., 2021). A case series investigated the efficacy of fosfomycin-containing regimens in patients with bacteremia caused by pan-drug-resistant *A. baumannii*. Patients who received the fosfomycin regimen had significantly better survival rates than those who did not, and lower doses of fosfomycin improved bacterial clearance in combination therapy (Assimakopoulos et al., 2023). Therefore, treatment modalities based on colistin or sulbactam combined with fosfomycin warrant further study.

### 2.9 Glycopeptide or lipopeptide antibiotics

#### 2.9.1 Vancomycin

An increasing number of studies have reported that glycopeptides and lipopeptides are active against MDR *A. baumannii*. The glycopeptide antibiotic vancomycin is effective in treating methicillin-resistant *Staphylococcus aureus* (MRSA) infections by inhibiting peptidoglycan synthesis in bacterial cell walls. The combination of colistin with this class of antibiotics is a unique potential treatment for MDR *A. baumannii* (Claeys et al., 2014; Pokorny and Almeida, 2021). Colistin acts on the anionic lipopolysaccharide of Gram-negative bacteria, enabling the macromolecule vancomycin to reach the action site of the cell wall and exert its bactericidal effect. In colistin-resistant *A. baumannii*, the absence of lipopolysaccharides may result in increased sensitivity of the strain to glycopeptide antibiotics (Claeys et al., 2014; Van Groesen et al., 2021).

Vancomycin combined with colistin exhibited a high level of synergistic and bactericidal activity *in vitro* using the checkerboard method, time sterilization curve, and *Galleria mellonella* moth model (O'Hara et al., 2013; Oliva et al., 2019; Shinohara et al., 2019). Moreover, combining colistin with vancomycin and rifampicin has been reported to successfully treat a case of ventilator-associated pneumonia caused by pan-resistant *A. baumannii* (Oliva et al., 2017). However, the efficacy of the combination of vancomycin and colistin in clinical applications is not consistent and certain reports are contradictory. A retrospective study compared the efficacy and safety of colistin alone and in combination with vancomycin in treating ventilator-associated pneumonia and bacteremia caused by CRAB. In the combined vancomycin group, similar clinical cure, microbial eradication, and mortality rates were observed, as well as a higher rate of acute kidney injury (55.2% vs 28%) (Garnacho-Montero et al., 2014). The same conclusion was reported in a study of 365 patients at Chiang Mai Hospital (Katip and Oberdorfer, 2021). Sarkar et al. tried to solve this dilemma using vancomycin derivatives, which promote autophagy and destroy the biofilm of *A. baumannii*, thereby quickly killing CRAB (Sarkar et al., 2020). Although this combination has shown promising prospects *in vitro*, both have nephrotoxic side effects. The results of *in vivo* studies are contradictory, and numerous objective data are required to confirm the efficacy and safety of this combination before clinical application.

#### 2.9.2 Daptomycin

The lipopeptide antibiotic daptomycin is often used as an option after second-line treatment has failed. It destroys bacterial cell membranes by reorganizing their structure, further affecting normal physiological activities such as cell wall and lipid syntheses and cellular respiration. The presence of lipopolysaccharide membranes in Gram-negative bacteria prevents daptomycin from binding to the cell membrane; however, when combined with colistin, it can enter the cell (Pokorny and Almeida, 2021; Ledger et al., 2022).

A pharmacokinetic model confirmed that the combination of daptomycin and colistin extended the survival time of mice infected with CRAB and effectively improved the therapeutic effect (Cirioni et al., 2016; Poulakou et al., 2019). Studies have confirmed the effectiveness of this protocol by conjugating selective siderophores with daptomycin (Ghosh et al., 2017). Although the results of this combination were promising both *in vivo* and *in vitro*, there are concerns regarding its clinical application. Currently, clinical validation is limited; thus, the efficacy and safety of this combination remain to be confirmed.

## 3 Future treatment options for CRAB infections

### 3.1 Bacteriophages

Bacteriophages are viruses that can infect various microorganisms. It kills pathogens without destroying the symbiotic flora and has strict host specificity. As early as the 20th century, humans have initiated the utilization of lytic phages, which exhibit rapid proliferation within bacteria and induce the prompt lysis of host bacteria (Tan et al., 2022). With the advent of the post-antibiotic era, researchers are focusing on phage therapy, which has attracted attention by researchers worldwide. Given the current difficulty in treating severe CRAB infections, bacteriophage therapy may be a potential alternative to current treatments (Isler et al., 2019).

In 2010, the first study to use lytic phages AB1 and AB2 against multi-resistant *A. baumannii* was reported (Lin et al., 2010). Jin et al. selected the lytic bacteriophage ZZ1, which can infect *A. baumannii*, for characterization and indicated that it has high antibacterial potential and practicability owing to its superior pH stability, heat resistance, and lytic spectrum (Jin et al., 2012). Thummeepak et al. (2016) cloned and overexpressed endolysin (LysABP-01) from *A. baumannii* bacteriophage ØABP-01. They demonstrated that this endolysin can hydrolyze the cell wall of *A. baumannii* and has synergistic antibacterial activity with colistin (Thummeepak et al., 2016). Numerous studies have demonstrated the successful use of phages in animals to cure CRAB infections; however, data on their use in humans remain limited (Kusradze et al., 2016; Regeimbal et al., 2016; Hua et al., 2017; Yin et al., 2017). Current approaches for related therapies include single-phage, phage cocktail, and phage-antibiotic combination therapies, phage-derived enzymes, and other recent advances in phage interventions. The successful treatment of CRAB infections with phages has been reported in several cases. For example, a 68-year-old patient with diabetes and necrotizing pancreatitis infected with *A. baumannii* was successfully treated with intravenous and percutaneous injections of phages into the abscess cavity (Schooley et al., 2017). Moreover, when combined with tigecycline and colistin (16 days of continuous aerosol administration), phages were used to successfully treat an 88-year-old patient with pneumonia caused by *A. baumannii* (Tan et al., 2021). Wu et al. conducted phage therapy (at 2 successive doses of 109 plaque-forming unit phages) in four patients with severe pneumonia caused by COVID-19 and CRAB pathogens, and the results revealed the therapeutic potential of phages in patients with severe pneumonia (Wu et al., 2021). Rao et al. reported that phage-antibiotic combination therapy was successfully used to treat an elderly patient with severe pneumonia caused by MDR *A. baumannii* (Rao et al., 2022).

However, various challenges remain regarding the widespread use of bacteriophages, such as the emergence of partially resistant bacteriophages, high diversity of pathogen genomes, limited host spectrum for clinical therapy, and efficacy of antibiotics in combination with bacteriophages. Therefore, evidence from *in vivo* studies and clinical trials is needed before bacteriophage therapy can be widely used clinically (Isler et al., 2019).

### 3.2 Antimicrobial peptides

Antimicrobial peptides (AMPs) are resistant to heat and acid–base interactions and are the first line of defense against biological hosts. In addition to their antimicrobial, anti-inflammatory, and anti-biofilm properties, numerous AMPs have immunomodulatory capabilities and are not prone to drug resistance, unlike conventional antibiotics (Krishnan et al., 2021). Huang et al. reported that when mouse models of pneumonia and peritonitis caused by CRAB pathogens were treated with cysteine-functionalized α-helical peptides, the symptoms of infection significantly improved (Huang et al., 2012). Pollini et al. reported that when combined with antibiotics, a new antimicrobial peptide, SET-M33, had synergistic antibacterial effects against both *A. baumannii* and *Klebsiella pneumoniae* (Pollini et al., 2017). The efficacy of the cyclic peptide cathelicidin-BF15-a4 against infections caused by MDR *A. baumannii* was demonstrated in a mouse septicemia model (Mwangi et al., 2019). Krishnan et al. also developed short 9-meric peptides that had therapeutic effects against CRAB infections. These peptides had low cytotoxicity and high selectivity (Krishnan et al., 2021).

Although AMPs have broad-spectrum bactericidal and immunomodulatory properties, their susceptibility to enzymatic degradation limits their clinical applications. AMPs could be candidates for the future treatment of CRAB; however, more basic research and clinical evidence are essential to further prove their antibacterial effects and safety.

## 4 Conclusions

In this review, the current and future treatment options for CRAB infections are discussed. The mechanisms of action, promises, perils, attention, and common combinations of antibacterial agents used for treating CRAB infections are summarized in **Table 1**. The mechanisms of action of antibiotics in treating CRAB infections are shown in **Figure 1**. With the increasing incidence of CRAB infections, realizing effective treatment drugs and combination plans has become a major challenge for clinicians. Although numerous studies have reported on the drug treatment of CRAB, clinical evidence is essential to confirm the effectiveness and safety of current clinical drugs and new drugs for CRAB. No single treatment option with an absolute advantage currently exists, and no consensus has emerged on the established therapeutic regimen for CRAB infections. At the time of initial treatment, high doses of ampicillin-sulbactam or tigecycline may be the best option. Polymyxins are limited by their dose and side effects, and new drugs such as durlobactam and cefiderocol have substantial therapeutic potential. SUL/DUR may be a preferable solution for patients with severe CRAB infections in the absence of better antimicrobial options, and cefiderocol may be a valuable alternative for treating serious CRAB infections. Moreover, bacteriophages and AMPs may serve as alternative treatment options in the future. A combination antimicrobial treatment may be more effective than a single regimen. For example, despite its significant nephrotoxicity, colistin is often used in combination with antimicrobials, such as tigecycline, ampicillin-sulbactam, meropenem, or fosfomycin. The IDSA has deemed high-dose ampicillin-sulbactam, which is typically combined with high-dose tigecycline, polymyxin, and other antibacterial agents, the backbone option for treating serious CRAB infections. A combination of meropenem, ampicillin/sulbactam, and polymyxin B for treating pneumonia and bloodstream infections caused by CRAB isolates is recommended by the SIDP. The ESCMID recommends the use of a combination of two *in vitro* active agents, including polymyxin, tigecycline, ampicillin/sulbactam, aminoglycoside, and high-dose meropenem, and to avoid colistin combined with meropenem or rifampicin. In summary, it is hoped that through a rational combination of drug use and the exploration of new therapeutic drugs, the effects of alleviating or preventing CRAB infections and reducing the length of hospital stays and mortality rates of patients can be achieved.

## Author contributions

SZ: Writing – original draft, Writing – review & editing. LD: Writing – original draft, Writing – review & editing. YQ: Writing – original draft, Writing – review & editing. XQ: Writing – original draft, Writing – review & editing. SW: Conceptualization, Data curation, Formal analysis, Funding acquisition, Investigation, Methodology, Project administration, Resources, Software, Supervision, Validation, Visualization, Writing – original draft, Writing – review & editing.

## References

[B1] Abdul-Mutakabbir J. C. Griffith N. C. Shields R. K. Tverdek F. P. Escobar Z. K. (2021). Contemporary perspective on the treatment of acinetobacter baumannii infections: insights from the society of infectious diseases pharmacists. Infect. Dis. Ther. 10, 2177–2202. doi: 10.1007/s40121-021-00541-4 34648177 PMC8514811

[B2] Alp E. Eren E. Elay G. Cevahir F. Esmaoğlu A. Rello J. (2017). Efficacy of loading dose of colistin in Acinetobacter baumannii ventilator-associated pneumonia. Infez. Med. 25, 311–319. 29286009

[B3] Al-Shaer M. Nazer L. H. Kherallah M. (2014). Rifampicin as adjunct to colistin therapy in the treatment of multidrug-resistant Acinetobacter baumannii. Ann. Pharmacother. 48, 766–771. doi: 10.1177/1060028014528306 24651164

[B4] Assimakopoulos S. F. Karamouzos V. Eleftheriotis G. Lagadinou M. Bartzavali C. Kolonitsiou F.. (2023). Acinetobacter baumanniiEfficacy of fosfomycin-containing regimens for treatment of bacteremia due to pan-drug resistant in critically ill patients: A case series study. Pathogens 12, 286. doi: 10.3390/pathogens12020286 36839558 PMC9961360

[B5] Assimakopoulos S. F. Karamouzos V. Lefkaditi A. Sklavou C. Kolonitsiou F. Christofidou M.. (2019). Triple combination therapy with high-dose ampicillin/sulbactam, high-dose tigecycline and colistin in the treatment of ventilator-associated pneumonia caused by pan-drug resistant Acinetobacter baumannii: a case series study. Infez. Med. 27, 11–16. 30882373

[B6] Aydemir H. Akduman D. Piskin N. Comert F. Horuz E. Terzi A.. (2013). Colistin vs. the combination of colistin and rifampicin for the treatment of carbapenem-resistant Acinetobacter baumannii ventilator-associated pneumonia. Epidemiol. Infect. 141, 1214–1222. doi: 10.1017/S095026881200194X 22954403 PMC9151808

[B7] Bae S. Kim M. C. Park S. J. Kim H. S. Sung H. Kim M. N.. (2016). *In Vitro* Synergistic Activity of Antimicrobial Agents in Combination against Clinical Isolates of Colistin-Resistant Acinetobacter baumannii. Antimicrob. Agents Chemother. 60, 6774–6779. doi: 10.1128/AAC.00839-16 27600048 PMC5075085

[B8] Band V. I. Hufnagel D. A. Jaggavarapu S. Sherman E. X. Wozniak J. E. Satola S. W.. (2019). Antibiotic combinations that exploit heteroresistance to multiple drugs effectively control infection. Nat. Microbiol. 4, 1627–1635. doi: 10.1038/s41564-019-0480-z 31209306 PMC7205309

[B9] Bartal C. Rolston K. V. Nesher L. (2022). Carbapenem-resistant acinetobacter baumannii: colonization, infection and current treatment options. Infect. Dis. Ther. 11, 683–694. doi: 10.1007/s40121-022-00597-w 35175509 PMC8960525

[B10] Bassetti M. Echols R. Matsunaga Y. Ariyasu M. Doi Y. Ferrer R.. (2021). Efficacy and safety of cefiderocol or best available therapy for the treatment of serious infections caused by carbapenem-resistant Gram-negative bacteria (CREDIBLE-CR): a randomised, open-label, multicentre, pathogen-focused, descriptive, phase 3 trial. Lancet Infect. Dis. 21, 226–240. doi: 10.1016/S1473-3099(20)30796-9 33058795

[B11] Bavaro D. F. Belati A. Diella L. Stufano M. Romanelli F. Scalone L.. (2021). Cefiderocol-based combination therapy for "Difficult-to-treat" Gram-negative severe infections: real-life case series and future perspectives. Antibiot. (Basel). 10, 652. doi: 10.3390/antibiotics10060652 PMC8227820 34072342

[B12] Bavaro D. F. Papagni R. Belati A. Diella L. De Luca A. Brindicci G.. (2023). Cefiderocol versus colistin for the treatment of carbapenem-resistant acinetobacter baumannii complex bloodstream infections: A retrospective, propensity-score adjusted, monocentric cohort study. Infect. Dis. Ther. 12, 2147–2163. doi: 10.1007/s40121-023-00854-6 37653122 PMC10505116

[B13] Beganovic M. Daffinee K. E. Luther M. K. LaPlante K. L. (2021). *In Vitro*Minocycline Alone and in Combination with Polymyxin B, Meropenem, and Sulbactam against Carbapenem-Susceptible and -Resistant Acinetobacter baumannii in an Pharmacodynamic Model. Antimicrob. Agents Chemother. 65, 10.1128/aac.01680-20. doi: 10.1128/AAC.01680-20 PMC8092495 33318006

[B14] Betrosian A. P. Frantzeskaki F. Xanthaki A. Douzinas E. E. (2008). Efficacy and safety of high-dose ampicillin/sulbactam vs. colistin as monotherapy for the treatment of multidrug resistant Acinetobacter baumannii ventilator-associated pneumonia. J. Infect. 56, 432–436. doi: 10.1016/j.jinf.2008.04.002 18501431

[B15] Chuang Y. C. Cheng C. Y. Sheng W. H. Sun H. Y. Wang J. T. Chen Y. C.. (2014). Effectiveness of tigecycline-based versus colistin- based therapy for treatment of pneumonia caused by multidrug-resistant Acinetobacter baumannii in a critical setting: a matched cohort analysis. BMC Infect. Dis. 14, 102. doi: 10.1186/1471-2334-14-102 24564226 PMC3936940

[B16] Cirioni O. Simonetti O. Pierpaoli E. Barucca A. Ghiselli R. Orlando F.. (2016). Colistin enhances therapeutic efficacy of daptomycin or teicoplanin in a murine model of multiresistant Acinetobacter baumannii sepsis. Diagn. Microbiol. Infect. Dis. 86, 392–398. doi: 10.1016/j.diagmicrobio.2016.09.010 27712928

[B17] Claeys K. C. Fiorvento A. D. Rybak M. J. (2014). A review of novel combinations of colistin and lipopeptide or glycopeptide antibiotics for the treatment of multidrug-resistant Acinetobacter baumannii. Infect. Dis. Ther. 3, 69–81. doi: 10.1007/s40121-014-0051-9 25475412 PMC4269621

[B18] Corcione S. De Benedetto I. Pinna S. M. Vita D. Lupia T. Montrucchio G.. (2022). Cefiderocol use in Gram negative infections with limited therapeutic options: Is combination therapy the key? J. Infect. Public Health 15, 975–979. doi: 10.1016/j.jiph.2022.07.006 35961239

[B19] De Pascale G. Lisi L. Ciotti G. M. P. Vallecoccia M. S. Cutuli S. L. Cascarano L.. (2020). Pharmacokinetics of high-dose tigecycline in critically ill patients with severe infections. Ann. Intensive Care 10, 94. doi: 10.1186/s13613-020-00715-2 32661791 PMC7357259

[B20] Durante-Mangoni E. Signoriello G. Andini R. Mattei A. De Cristoforo M. Murino P.. (2013). Colistin and rifampicin compared with colistin alone for the treatment of serious infections due to extensively drug-resistant Acinetobacter baumannii: a multicenter, randomized clinical trial. Clin. Infect. Dis. 57, 349–358. doi: 10.1093/cid/cit253 23616495

[B21] Falcone M. Tiseo G. Leonildi A. Della Sala L. Vecchione A. Barnini S.. (2022). Cefiderocol- compared to colistin-based regimens for the treatment of severe infections caused by carbapenem-resistant acinetobacter baumannii. Antimicrob. Agents Chemother. 66, e0214221. doi: 10.1128/aac.02142-21 35311522 PMC9112922

[B22] Findlay J. Poirel L. Bouvier M. Nordmann P. (2022). *In vitro* activity of sulbactam-durlobactam against carbapenem-resistant Acinetobacter baumannii and mechanisms of resistance. J. Glob. Antimicrob. Resist. 30, 445–450. doi: 10.1016/j.jgar.2022.05.011 35618210

[B23] Garnacho-Montero J. Amaya-Villar R. Gutiérrez-Pizarraya A. Espejo-Gutiérrez de Tena E. Artero-González M. L. Corcia-Palomo Y.. (2014). Clinical efficacy and safety of the combination of colistin plus vancomycin for the treatment of severe infections caused by carbapenem-resistant Acinetobacter baumannii. Chemotherapy 59, 225–231. doi: 10.1159/000356004 24356297

[B24] Gavaghan V. Miller J. L. Dela-Pena J. (2023). Case series of cefiderocol for salvage therapy in carbapenem-resistant Gram-negative infections. Infection 51, 475–482. doi: 10.1007/s15010-022-01933-5 36201152 PMC9540105

[B25] Ghosh M. Miller P. A. Möllmann U. Claypool W. D. Schroeder V. A. Wolter W. R.. (2017). Targeted antibiotic delivery: selective siderophore conjugation with daptomycin confers potent activity against multidrug resistant Acinetobacter baumannii both *in vitro* and *in vivo* . J. Med. Chem. 60, 4577–4583. doi: 10.1021/acs.jmedchem.7b00102 28287735

[B26] Goff D. A. Bauer K. A. Mangino J. E. (2014). Bad bugs need old drugs: a stewardship program's evaluation of minocycline for multidrug-resistant Acinetobacter baumannii infections. Clin. Infect. Dis. null, S381–S387. doi: 10.1093/cid/ciu593 25371514

[B27] Greig S. L. Scott L. J. (2016). Intravenous minocycline: A review in acinetobacter infections. Drugs 76, 1467–1476. doi: 10.1007/s40265-016-0636-6 27573640

[B28] Holger D. J. Kunz Coyne A. J. Zhao J. J. Sandhu A. Salimnia H. Rybak M. J. (2022). Acinetobacter baumanniiNovel combination therapy for extensively drug-resistant necrotizing pneumonia complicated by empyema: A case report. Open Forum Infect. Dis. 9, ofac092. doi: 10.1093/ofid/ofac092 35350174 PMC8946682

[B29] Hsueh S. C. Lee Y. J. Huang Y. T. Liao C. H. Tsuji M. Hsueh P. R. (2019). *In vitro* activities of cefiderocol, ceftolozane/tazobactam, ceftazidime/avibactam and other comparative drugs against imipenem-resistant Pseudomonas aeruginosa and Acinetobacter baumannii, and Stenotrophomonas maltophilia, all associated with bloodstream infections in Taiwan. J. Antimicrob. Chemother. 74, 380–386. doi: 10.1093/jac/dky425 30357343

[B30] Hua Y. Luo T. Yang Y. Dong D. Wang R. Wang Y.. (2017). Acinetobacter baumanniiPhage therapy as a promising new treatment for lung infection caused by carbapenem-resistant in mice. Front. Microbiol. 8. doi: 10.3389/fmicb.2017.02659 PMC5767256 29375524

[B31] Huang Y. Wiradharma N. Xu K. Ji Z. Bi S. Li L.. (2012). Cationic amphiphilic alpha-helical peptides for the treatment of carbapenem-resistant Acinetobacter baumannii infection. Biomaterials 33, 8841–8847. doi: 10.1016/j.biomaterials.2012.08.026 22925814

[B32] Isler B. Doi Y. Bonomo R. A. Paterson D. L. (2019). Acinetobacter baumanniiNew Treatment Options against Carbapenem-Resistant Infections. Antimicrob. Agents Chemother. 63, 10.1128/aac.01110-18. doi: 10.1128/AAC.01110-18 PMC6325237 30323035

[B33] Jin J. Li Z. J. Wang S. W. Wang S. M. Huang D. H. Li Y. H.. (2012). Isolation and characterization of ZZ1, a novel lytic phage that infects Acinetobacter baumannii clinical isolates. BMC Microbiol. 12, 156. doi: 10.1186/1471-2180-12-156 22838726 PMC3438129

[B34] Jung S. Y. Lee S. H. Lee S. Y. Yang S. Noh H. Chung E. K.. (2017). Antimicrobials for the treatment of drug-resistant Acinetobacter baumannii pneumonia in critically ill patients: a systemic review and Bayesian network meta-analysis. Crit. Care 21, 319. doi: 10.1186/s13054-017-1916-6 29262831 PMC5738897

[B35] Karakonstantis S. Kritsotakis E. I. Gikas A. (2020). Treatment options for K. pneumoniae, P. aeruginosa and A. baumannii co-resistant to carbapenems, aminoglycosides, polymyxins and tigecycline: an approach based on the mechanisms of resistance to carbapenems. Infection 48, 835–851. doi: 10.1007/s15010-020-01520-6 32875545 PMC7461763

[B36] Karruli A. Migliaccio A. Pournaras S. Durante-Mangoni E. Zarrilli R. (2023). Acinetobacter baumanniiCefiderocol and Sulbactam-Durlobactam against Carbapenem-Resistant. Antibiot. (Basel). 12, 1729. doi: 10.3390/antibiotics12121729 PMC10740486 38136764

[B37] Katip W. Meechoui M. Thawornwittayakom P. Chinwong D. Oberdorfer P. (2019). Efficacy and safety of high loading dose of colistin in multidrug-resistant Acinetobacter baumannii: a prospective cohort study. J. Intensive Care Med. 34, 996–1002. doi: 10.1177/0885066617725694 28820037

[B38] Katip W. Oberdorfer P. (2021). Clinical efficacy and nephrotoxicity of colistin alone versus colistin plus vancomycin in critically ill patients infected with carbapenem-resistant Acinetobacter baumannii: a propensity score-matched analysis. Pharmaceutics 13, 162. doi: 10.3390/pharmaceutics13020162 33530483 PMC7912140

[B39] Katip W. Oberdorfer P. Kasatpibal N. (2022). Effectiveness and nephrotoxicity of loading dose colistin–meropenem versus loading dose colistin–imipenem in the treatment of carbapenem-resistant Acinetobacter baumannii infection. Pharmaceutics 14, 1266. doi: 10.3390/pharmaceutics14061266 35745838 PMC9228626

[B40] Katip W. Uitrakul S. Oberdorfer P. (2017). Clinical outcomes and nephrotoxicity of colistin loading dose for treatment of extensively drug-resistant Acinetobacter baumannii in cancer patients. Infect. Drug Resist. 10, 293–298. doi: 10.2147/IDR.S144314 28919792 PMC5593398

[B41] Katip W. Uitrakul S. Oberdorfer P. (2021). Clinical efficacy and nephrotoxicity of the loading dose colistin for the treatment of carbapenem-resistant Acinetobacter baumannii in critically ill patients. Pharmaceutics 14, 31. doi: 10.3390/pharmaceutics14010031 35056926 PMC8780224

[B42] Kaye K. S. Shorr A. F. Wunderink R. G. Du B. Poirier G. E. Rana K.. (2023). Efficacy and safety of sulbactam-durlobactam versus colistin for the treatment of patients with serious infections caused by Acinetobacter baumannii-calcoaceticus complex: a multicentre, randomised, active-controlled, phase 3, non-inferiority clinical trial (ATTACK). Lancet Infect. Dis. 23, 1072–1084. doi: 10.1016/S1473-3099(23)00184-6 37182534

[B43] Kengkla K. Kongpakwattana K. Saokaew S. Apisarnthanarak A. Chaiyakunapruk N. (2018). Comparative efficacy and safety of treatment options for MDR and XDR Acinetobacter baumannii infections: a systematic review and network meta-analysis. J. Antimicrob. Chemother. 73, 22–32. doi: 10.1093/jac/dkx368 29069421

[B44] Khalili H. Shojaei L. Mohammadi M. Beigmohammadi M. T. Abdollahi A. Doomanlou M. (2018). Meropenem/colistin versus meropenem/ampicillin-sulbactam in the treatment of carbapenem-resistant pneumonia. J. Comp. Eff. Res. 7, 901–911. doi: 10.2217/cer-2018-0037 30192166

[B45] Kollef M. Dupont H. Greenberg D. E. Viale P. Echols R. Yamano Y.. (2023). Prospective role of cefiderocol in the management of carbapenem-resistant Acinetobacter baumannii infections: Review of the evidence. Int. J. Antimicrob. Agents 62, 106882. doi: 10.1016/j.ijantimicag.2023.106882 37301312

[B46] Krishnan M. Choi J. Jang A. Yoon Y. K. Kim Y. (2021). Acinetobacter baumanniiAntiseptic 9-Meric Peptide with Potency against Carbapenem-Resistant Infection. Int. J. Mol. Sci. 22, 12520. doi: 10.3390/ijms222212520 34830401 PMC8621208

[B47] Kusradze I. Karumidze N. Rigvava S. Dvalidze T. Katsitadze M. Amiranashvili I.. (2016). Acinetobacter baumanniiCharacterization and testing the efficiency of phage as an antibacterial agent. Front. Microbiol. 7. doi: 10.3389/fmicb.2016.01590 PMC5047890 27757110

[B48] Ledger E. V. Sabnis A. Edwards A. M. (2022). Polymyxin and lipopeptide antibiotics: membrane-targeting drugs of last resort. Microbiol. (Reading). 168, 1136. doi: 10.1099/mic.0.001136 PMC8941995 35118938

[B49] Lenhard J. R. Smith N. M. Bulman Z. P. Tao X. Thamlikitkul V. Shin B. S. (2017). High-dose ampicillin-sulbactam combinations combat polymyxin-resistant acinetobacter baumannii in a hollow-fiber infection model. Antimicrob. Agents Chemother. 61, Undefined. doi: 10.1128/AAC.01268-16 PMC5328540 28052852

[B50] Liang C. A. Lin Y. C. Lu P. L. Chen H. C. Chang H. L. Sheu C. C. (2018). Antibiotic strategies and clinical outcomes in critically ill patients with pneumonia caused by carbapenem-resistant Acinetobacter baumannii. Clin. Microbiol. Infect. 24, 908.e1–908.e7. doi: 10.1016/j.cmi.2017.10.033 29108947

[B51] Liebchen U. Paal M. Jung J. Schroeder I. Frey L. Zoller M.. (2020). Acinetobacter baumannii -Therapeutic drug monitoring-guided high dose meropenem therapy of a multidrug resistant A case report. Respir. Med. Case Rep. 29, 100966. doi: 10.1016/j.rmcr.2019.100966 31871885 PMC6909205

[B52] Lin N. T. Chiou P. Y. Chang K. C. Chen L. K. Lai M. J. (2010). Isolation and characterization of phi AB2: a novel bacteriophage of Acinetobacter baumannii. Res. Microbiol. 161, 308–314. doi: 10.1016/j.resmic.2010.03.007 20385229

[B53] Liu J. Shu Y. Zhu F. Feng B. Zhang Z. Liu L.. (2021). Comparative efficacy and safety of combination therapy with high-dose sulbactam or colistin with additional antibacterial agents for multiple drug-resistant and extensively drug-resistant Acinetobacter baumannii infections: A systematic review and network meta-analysis. J. Glob. Antimicrob. Resist. 24, 136–147. doi: 10.1016/j.jgar.2020.08.021 32889142

[B54] Mabayoje D. A. NicFhogartaigh C. Cherian B. P. Tan M. G. M. Wareham D. W. (2021). Acinetobacter baumanniiCompassionate use of cefiderocol for carbapenem-resistant prosthetic joint infection. JAC. Antimicrob. Resist. 3, i21–i24. doi: 10.1093/jacamr/dlab055 34223152 PMC8251250

[B55] Marino A. Stracquadanio S. Campanella E. Munafò A. Gussio M. Ceccarelli M.. (2022). Intravenous fosfomycin: A potential good partner for cefiderocol. Clinical experience and considerations. Antibiot. (Basel). 12, Undefined. doi: 10.3390/antibiotics12010049 PMC9854867 36671250

[B56] Mazzitelli M. Gregori D. Sasset L. Trevenzoli M. Scaglione V. Lo Menzo S.. (2023). Acinetobacter baumanniiCefiderocol-Based versus Colistin-Based Regimens for Severe Carbapenem-Resistant Infections: A Propensity Score-Weighted, Retrospective Cohort Study during the First Two Years of the COVID-19 Pandemic. Microorganisms 11, 984. doi: 10.3390/microorganisms11040984 37110408 PMC10146662

[B57] Moni M. Sudhir A. S. Dipu T. S. Mohamed Z. Prabhu B. P. Edathadathil F.. (2020). Clinical efficacy and pharmacokinetics of colistimethate sodium and colistin in critically ill patients in an Indian hospital with high endemic rates of multidrug-resistant Gram-negative bacterial infections: A prospective observational study. Int. J. Infect. Dis. 100, 497–506. doi: 10.1016/j.ijid.2020.08.010 32781161

[B58] Morrissey I. Olesky M. Hawser S. Lob S. H. Karlowsky J. A. Corey G. R.. (2020). *In vitro* activity of eravacycline against gram-negative bacilli isolated in clinical laboratories worldwide from 2013 to 2017. Antimicrob. Agents Chemother. 64, 10.1128/aac.01699-19. doi: 10.1128/AAC.01699-19 PMC7038303 31843999

[B59] Mueller L. Masseron A. Prod’Hom G. Galperine T. Greub G. Poirel L.. (2019). Phenotypic, biochemical and genetic analysis of KPC-41, a KPC-3 variant conferring resistance to ceftazidime-avibactam and exhibiting reduced carbapenemase activity. Antimicrob. Agents Chemother. 63, 10.1128/aac.01111-19. doi: 10.1128/AAC.01111-19 PMC6879233 31527032

[B60] Murray C. J. Ikuta K. S. Sharara F. Swetschinski L. Aguilar G. R. Gray A.. (2022). Global burden of bacterial antimicrobial resistance in 2019: a systematic analysis. Lancet 399, 629–655. doi: 10.1016/S0140-6736(21)02724-0 35065702 PMC8841637

[B61] Mwangi J. Yin Y. Wang G. Yang M. Li Y. Zhang Z.. (2019). Pseudomonas aeruginosaThe antimicrobial peptide ZY4 combats multidrug-resistant and infection. Proc. Natl. Acad. Sci. U.S.A. 116, 26516–26522. doi: 10.1073/pnas.1909585117 31843919 PMC6936460

[B62] Ni W. Han Y. Zhao J. Wei C. Cui J. Wang R.. (2016). Tigecycline treatment experience against multidrug-resistant Acinetobacter baumannii infections: a systematic review and meta-analysis. Int. J. Antimicrob. Agents 47, 107–116. doi: 10.1016/j.ijantimicag.2015.11.011 26742726

[B63] Niederman M. S. Alder J. Bassetti M. Boateng F. Cao B. Corkery K.. (2020). Inhaled amikacin adjunctive to intravenous standard-of-care antibiotics in mechanically ventilated patients with Gram-negative pneumonia (INHALE): a double-blind, randomised, placebo-controlled, phase 3, superiority trial. Lancet Infect. Dis. 20, 330–340. doi: 10.1016/S1473-3099(19)30574-2 31866328

[B64] Niu T. Luo Q. Li Y. Zhou Y. Yu W. Xiao Y. (2019). Acinetobacter baumanniiComparison of Tigecycline or Cefoperazone/Sulbactam therapy for bloodstream infection due to Carbapenem-resistant. Antimicrob. Resist. Infect. Control. 8, 52. doi: 10.1186/s13756-019-0502-x 30886705 PMC6404342

[B65] Noel A. R. Attwood M. Bowker K. E. MacGowan A. P. (2021). *In vitro* pharmacodynamics of omadacycline against Escherichia coli and Acinetobacter baumannii. J. Antimicrob. Chemother. 76, 667–670. doi: 10.1093/jac/dkaa508 33294925

[B66] O'Donnell J. N. Putra V. Lodise T. P. (2021). Treatment of patients with serious infections due to carbapenem-resistant Acinetobacter baumannii: How viable are the current options? Pharmacotherapy 41, 762–780. doi: 10.1002/phar.2607 34170571

[B67] O'Hara J. A. Ambe L. A. Casella L. G. Townsend B. M. Pelletier M. R. Ernst R. K.. (2013). Activities of vancomycin-containing regimens against colistin-resistant Acinetobacter baumannii clinical strains. Antimicrob. Agents Chemother. 57, 2103–2108. doi: 10.1128/AAC.02501-12 PMC3632926 23422916

[B68] Oliva A. Cipolla A. Vullo V. Venditti M. Mastroianni C. M. Falcone M. (2017). Clinical and *in vitro* efficacy of colistin plus vancomycin and rifampin against colistin-resistant Acinetobacter baumannii causing ventilator-associated pneumonia. New Microbiol. 40, 205–207. 28675246

[B69] Oliva A. Garzoli S. De Angelis M. Marzuillo C. Vullo V. Mastroianni C. M.. (2019). *In-vitro* evaluation of different antimicrobial combinations with and without colistin against carbapenem-resistant Acinetobacter baumannii. Molecules 24, 886. doi: 10.3390/molecules24050886 30832412 PMC6429394

[B70] Oliveira P. R. Carvalho V. C. Saconi E. S. Leonhardt M. C. Kojima K. E. Santos J. S.. (2020). Acinetobacter baumanniiTigecycline versus colistin in the treatment of carbapenem-resistant complex osteomyelitis. J. Bone Jt. Infect. 5, 60–66. doi: 10.7150/jbji.42448 32455096 PMC7242406

[B71] Park H. J. Cho J. H. Kim H. J. Han S. H. Jeong S. H. Byun M. K. (2019). Colistin monotherapy versus colistin/rifampicin combination therapy in pneumonia caused by colistin-resistant Acinetobacter baumannii: A randomised controlled trial. J. Glob. Antimicrob. Resist. 17, 66–71. doi: 10.1016/j.jgar.2018.11.016 30476654

[B72] Park J. M. Yang K. S. Chung Y. S. Lee K. B. Kim J. Y. Kim S. B.. (2021). Clinical outcomes and safety of meropenem–colistin versus meropenem–tigecycline in patients with carbapenem-resistant Acinetobacter baumannii pneumonia. Antibiotics 10, 903. doi: 10.3390/antibiotics10080903 34438953 PMC8388669

[B73] Pascale R. Pasquini Z. Bartoletti M. Caiazzo L. Fornaro G. Bussini L.. (2021). Acinetobacter baumanniiCefiderocol treatment for carbapenem-resistant infection in the ICU during the COVID-19 pandemic: a multicentre cohort study. JAC. Antimicrob. Resist. 3, dlab174. doi: 10.1093/jacamr/dlab174 34806011 PMC8599913

[B74] Paul M. Carrara E. Retamar P. Tängdén T. Bitterman R. Bonomo R. A.. (2022). European Society of Clinical Microbiology and Infectious Diseases (ESCMID) guidelines for the treatment of infections caused by multidrug-resistant Gram-negative bacilli (endorsed by European society of intensive care medicine). Clin. Microbiol. Infect. 28, 521–547. doi: 10.1016/j.cmi.2021.11.025 34923128

[B75] Penwell W. F. Shapiro A. B. Giacobbe R. A. Gu R. F. Gao N. Thresher J.. (2015). Molecular mechanisms of sulbactam antibacterial activity and resistance determinants in Acinetobacter baumannii. Antimicrob. Agents Chemother. 59, 1680–1689. doi: 10.1128/AAC.04808-14 25561334 PMC4325763

[B76] Pokorny A. Almeida P. F. (2021). The antibiotic peptide daptomycin functions by reorganizing the membrane. J. Membr. Biol. 254, 97–108. doi: 10.1007/s00232-021-00175-0 33620544

[B77] Pollini S. Brunetti J. Sennati S. Rossolini G. M. Bracci L. Pini A.. (2017). Synergistic activity profile of an antimicrobial peptide against multidrug-resistant and extensively drug-resistant strains of Gram-negative bacterial pathogens. J. Pept. Sci. 23, 329–333. doi: 10.1002/psc.2978 28176481

[B78] Poulakou G. Renieris G. Sabrakos L. Zarkotou O. Themeli-Digalaki K. Perivolioti E.. (2019). Daptomycin as adjunctive treatment for experimental infection by Acinetobacter baumannii with resistance to colistin. Int. J. Antimicrob. Agents 53, 190–194. doi: 10.1016/j.ijantimicag.2018.10.024 30391645

[B79] Rao S. Betancourt-Garcia M. Kare-Opaneye Y. O. Swierczewski B. E. Bennett J. W. Horne B. A.. (2022). Critically ill patient with multidrug-resistant acinetobacter baumannii respiratory infection successfully treated with intravenous and nebulized bacteriophage therapy. Antimicrob. Agents Chemother. 66, e0082421. doi: 10.1128/AAC.00824-21 34662188 PMC8765271

[B80] Regeimbal J. M. Jacobs A. C. Corey B. W. Henry M. S. Thompson M. G. Pavlicek R. L.. (2016). Personalized therapeutic cocktail of wild environmental phages rescues mice from acinetobacter baumannii wound infections. Antimicrob. Agents Chemother. 60, 5806–5816. doi: 10.1128/AAC.02877-15 27431214 PMC5038255

[B81] Ritchie D. J. Garavaglia-Wilson A. (2014). A review of intravenous minocycline for treatment of multidrug-resistant Acinetobacter infections. Clin. Infect. Dis. 59, S374–S380. doi: 10.1093/cid/ciu613 25371513

[B82] Rodríguez C. H. Barberis C. Nastro M. Bombicino K. Granados G. Vay C.. (2012). Impact of heteroresistance to colistin in meningitis caused by Acinetobacter baumannii. J. Infect. 64, 119–121. doi: 10.1016/j.jinf.2011.10.007 22044781

[B83] Russo A. Bassetti M. Bellelli V. Bianchi L. Marincola Cattaneo F. Mazzocchetti S.. (2021). Efficacy of a fosfomycin-containing regimen for treatment of severe pneumonia caused by multidrug-resistant acinetobacter baumannii: A prospective, observational study. Infect. Dis. Ther. 10, 187–200. doi: 10.1007/s40121-020-00357-8 33068255 PMC7568458

[B84] Sarkar P. Samaddar S. Ammanathan V. Yarlagadda V. Ghosh C. Shukla M.. (2020). Vancomycin derivative inactivates carbapenem-resistant Acinetobacter baumannii and induces autophagy. ACS Chem. Biol. 15, 884–889. doi: 10.1021/acschembio.0c00091 32195571

[B85] Schooley R. T. Biswas B. Gill J. J. Hernandez-Morales A. Lancaster J. Lessor L.. (2017). Development and use of personalized bacteriophage-based therapeutic cocktails to treat a patient with a disseminated resistant Acinetobacter baumannii infection. Antimicrob. Agents Chemother. 61, 10–1128. doi: 10.1128/AAC.00954-17 PMC5610518 28807909

[B86] Scott C. J. Zhu E. Jayakumar R. A. Shan G. Viswesh V. (2022). VersusEfficacy of eravacycline best previously available therapy for adults with pneumonia due to difficult-to-treat resistant (DTR). Ann. Pharmacother. 56, 1299–1307. doi: 10.1177/10600280221085551 35511209

[B87] Seifert H. Blondeau J. Lucassen K. Utt E. A. (2022). Global update on the *in vitro* activity of tigecycline and comparators against isolates of Acinetobacter baumannii and rates of resistant phenotypes, (2016-2018). J. Glob. Antimicrob. Resist. 31, 82–89. doi: 10.1016/j.jgar.2022.08.002 35948242

[B88] Seok H. Choi W. S. Lee S. Moon C. Park D. W. Song J. Y.. (2021). What is the optimal antibiotic treatment strategy for carbapenem-resistant Acinetobacter baumannii (CRAB)? A multicentre study in Korea. J. Glob. Antimicrob. Resist. 24, 429–439. doi: 10.1016/j.jgar.2021.01.018 33571708

[B89] Shapiro A. B. (2017). Kinetics of sulbactam hydrolysis by β-lactamases, and kinetics of β-lactamase inhibition by sulbactam. Antimicrob. Agents Chemother. 61, lbactam. Antimicrob Agents Chemother 61:10.1128/aac.01612-17. doi: 10.1128/AAC.01612-17 PMC5700308 28971872

[B90] Shields R. K. Paterson D. L. Tamma P. D. (2023). Navigating available treatment options for carbapenem-resistant acinetobacter baumannii-calcoaceticus complex infections. Clin. Infect. Dis. 76, S179–S193. doi: 10.1093/cid/ciad094 37125467 PMC10150276

[B91] Shinohara D. R. Menegucci T. C. Fedrigo N. H. Migliorini L. B. Carrara-Marroni F. E. Maria dos Anjos M.. (2019). Synergistic activity of polymyxin B combined with vancomycin against carbapenem-resistant and polymyxin-resistant Acinetobacter baumannii: First *in vitro* study. J. Med. Microbiol. 68, 309–315. doi: 10.1099/jmm.0.000920 30663954

[B92] Sirijatuphat R. Thamlikitkul V. (2014). Preliminary study of colistin versus colistin plus fosfomycin for treatment of carbapenem-resistant Acinetobacter baumannii infections. Antimicrob. Agents Chemother. 58, 5598–5601. doi: 10.1128/AAC.02435-13 24982065 PMC4135862

[B93] Tacconelli E. Carrara E. Savoldi A. Harbarth S. Mendelson M. Monnet D. L.. (2018). Discovery, research, and development of new antibiotics: the WHO priority list of antibiotic-resistant bacteria and tuberculosis. Lancet Infect. Dis. 18, 318–327. doi: 10.1016/S1473-3099(17)30753-3 29276051

[B94] Tamma P. D. Aitken S. L. Bonomo R. A. Mathers A. J. Van Duin D. Clancy C. J. (2022a). Infectious Diseases Society of America 2022 Guidance on the Treatment of Extended-Spectrum β-lactamase Producing Enterobacterales (ESBL-E), Carbapenem-Resistant Enterobacterales (CRE), and Pseudomonas aeruginosa with Difficult-to-Treat Resistance (DTR-P. aeruginosa). Clin. Infect. Dis. 75, 187–212. doi: 10.1093/cid/ciac268 35439291 PMC9890506

[B95] Tamma P. D. Aitken S. L. Bonomo R. A. Mathers A. J. Van Duin D. Clancy C. J. (2022b). Infectious Diseases Society of America Guidance on the Treatment of AmpC β-Lactamase-Producing Enterobacterales, Carbapenem-Resistant Acinetobacter baumannii, and Stenotrophomonas maltophilia Infections. Clin. Infect. Dis. 74, 2089–2114. doi: 10.1093/cid/ciab1013 34864936

[B96] Tamma P. D. Aitken S. L. Bonomo R. A. Mathers A. J. van Duin D. Clancy C. J. (2023). Infectious diseases society of america 2023 guidance on the treatment of antimicrobial resistant gram-negative infections. Clin. Infect. Dis., ciad428. doi: 10.1093/cid/ciad428 37463564

[B97] Tan X. Chen H. Zhang M. Zhao Y. Jiang Y. Liu X.. (2021). Clinical experience of personalized phage therapy against carbapenem-resistant Acinetobacter baumannii lung infection in a patient with chronic obstructive pulmonary disease. Front. Cell Infect. Microbiol. 11. doi: 10.3389/fcimb.2021.631585 PMC7952606 33718279

[B98] Tan Y. Su J. Fu M. Zhang H. Zeng H. (2022). Acinetobacter baumanniiRecent advances in phage-based therapeutics for multi-drug resistant. Bioengineering. (Basel). 10, 35. doi: 10.3390/bioengineering10010035 36671607 PMC9855029

[B99] Thummeepak R. Kitti T. Kunthalert D. Sitthisak S. (2016). Enhanced antibacterial activity of acinetobacter baumannii bacteriophage ØABP-01 endolysin (LysABP-01) in combination with colistin. Front. Microbiol. 7. doi: 10.3389/fmicb.2016.01402 PMC5013039 27656173

[B100] Tiseo G. Giordano C. Leonildi A. Riccardi N. Galfo V. Limongi F.. (2023). Acinetobacter baumanniiSalvage therapy with sulbactam/durlobactam against cefiderocol-resistant in a critically ill burn patient: clinical challenges and molecular characterization. JAC. Antimicrob. Resist. 5, dlad078. doi: 10.1093/jacamr/dlad078 37325251 PMC10265591

[B101] Tsuji B. T. Pogue J. M. Zavascki A. P. Paul M. Daikos G. L. Forrest A.. (2019). International consensus guidelines for the optimal use of the polymyxins: endorsed by the american college of clinical pharmacy (ACCP), european society of clinical microbiology and infectious diseases (ESCMID), infectious diseases society of america (IDSA), international society for anti-infective pharmacology (ISAP), society of critical care medicine (SCCM), and society of infectious diseases pharmacists (SIDP). Pharmacotherapy 39, 10–39. doi: 10.1002/phar.2209 30710469 PMC7437259

[B102] US Food and Drug Administration. (2023). Highlights of Prescribing Information: Xacduro (sulbactam/durlobactam). Available online at: https://www.accessdata.fda.gov/drugsatfda_docs/label/2023/216974Orig1s000Correctedlbl.pdf (Accessed November 12, 2023).

[B103] US Food and Drug Administration. (2019). Highlights of Prescribing Information: Fetroja (Cefiderocol). Available online at: https://www.accessdata.fda.gov/drugsatfda_docs/label/2019/209445s000lbl.pdf.

[B104] Van Groesen E. Slingerland C. J. Innocenti P. Mihajlovic M. Masereeuw R. Martin N. I. (2021). Vancomyxins: Vancomycin-polymyxin nonapeptide conjugates that retain anti-Gram-positive activity with enhanced potency against Gram-negative strains. ACS Infect. Dis. 7, 2746–2754. doi: 10.1021/acsinfecdis.1c00318 34387988 PMC8438664

[B105] Vardakas K. Z. Voulgaris G. L. Samonis G. Falagas M. E. (2018). Inhaled colistin monotherapy for respiratory tract infections in adults without cystic fibrosis: a systematic review and meta-analysis. Int. J. Antimicrob. Agents 51, 1–9. doi: 10.1016/j.ijantimicag.2017.05.016 28669836

[B106] Wagenlehner F. Lucenteforte E. Pea F. Soriano A. Tavoschi L. Steele V. R.. (2021). Systematic review on estimated rates of nephrotoxicity and neurotoxicity in patients treated with polymyxins. Clin. Microbiol. Infect. 27, 671–686. doi: 10.1016/j.cmi.2020.12.009 33359542

[B107] Wong D. Nielsen T. B. Bonomo R. A. Pantapalangkoor P. Luna B. Spellberg B. (2017). Clinical and Pathophysiological overview of acinetobacter infections: a century of challenges. Clin. Microbiol. Rev. 30, 409–447. doi: 10.1128/CMR.00058-16 27974412 PMC5217799

[B108] Wu N. Dai J. Guo M. Li J. Zhou X. Li F.. (2021). Acinetobacter baumanniiPre-optimized phage therapy on secondary infection in four critical COVID-19 patients. Emerg. Microbes Infect. 10, 612–618. doi: 10.1080/22221751.2021.1902754 33703996 PMC8032346

[B109] Yamano Y. Takemura M. Anan N. Nakamura R. Echols R. (2020). Synergistic effect of cefiderocol with other antibiotics against PER-producing Acinetobacter baumannii isolates from the multinational SIDERO-WT studies. Open Forum Infect. Dis. 7, S805. doi: 10.1093/ofid/ofaa439.1806

[B110] Yin S. Huang G. Zhang Y. Jiang B. Yang Z. Dong Z.. (2017). Phage abp1 rescues human cells and mice from infection by pan-drug resistant acinetobacter baumannii. Cell Physiol. Biochem. 44, 2337–2345. doi: 10.1159/000486117 29258062

[B111] Zaidan N. Hornak J. P. Reynoso D. (2021). Extensively drug-resistant acinetobacter baumannii nosocomial pneumonia successfully treated with a novel antibiotic combination. Antimicrob. Agents Chemother. 65, e0092421. doi: 10.1128/AAC.00924-21 34370576 PMC8522738

[B112] Zeng M. Xia J. Zong Z. Shi Y. Ni Y. Hu F.. (2023). Guidelines for the diagnosis, treatment, prevention and control of infections caused by carbapenem-resistant gram-negative bacilli. J. Microbiol. Immunol. Infect. 56, 653–671. doi: 10.1016/j.jmii.2023.01.017 36868960

[B113] Zha L. Pan L. Guo J. French N. Villanueva E. V. Tefsen B. (2020). Effectiveness and safety of high dose tigecycline for the treatment of severe infections: A systematic review and meta-analysis. Adv. Ther. 37, 1049–1064. doi: 10.1007/s12325-020-01235-y 32006240 PMC7223407

